# New methods to unveil host-microbe interaction mechanisms along the microbiota-gut-brain-axis

**DOI:** 10.1080/19490976.2024.2351520

**Published:** 2024-05-08

**Authors:** Habibullah Moradian, Tristan Gabriel, Mathilde Barrau, Xavier Roblin, Stéphane Paul

**Affiliations:** a CIRI – Centre International de Recherche en Infectiologie, Team GIMAP, Univ Lyon, Université Claude Bernard Lyon 1, Saint-Etienne, France; b CIC 1408 Inserm Vaccinology, University Hospital of Saint-Etienne, Saint-Etienne, France; c Immunology Department, University Hospital of Saint-Etienne, Saint-Etienne, France

**Keywords:** Microbiota-gut-brain, chip, model, preclinical, microbiota

## Abstract

Links between the gut microbiota and human health have been supported throughout numerous studies, such as the development of neurological disease disorders. This link is referred to as the “microbiota-gut-brain axis” and is the focus of an emerging field of research. Microbial-derived metabolites and gut and neuro-immunological metabolites regulate this axis in health and many diseases. Indeed, assessing these signals, whether induced by microbial metabolites or neuro-immune mediators, could significantly increase our knowledge of the microbiota-gut-brain axis. However, this will require the development of appropriate techniques and potential models. Methods for studying the induced signals originating from the microbiota remain crucial in this field. This review discusses the methods and techniques available for studies of microbiota-gut-brain interactions. We highlight several much-debated elements of these methodologies, including the widely used *in vivo* and *in vitro* models, their implications, and perspectives in the field based on a systematic review of PubMed. Applications of various animal models (zebrafish, mouse, canine, rat, rabbit) to microbiota-gut-brain axis research with practical examples of *in vitro* methods and innovative approaches to studying gut-brain communications are highlighted. In particular, we extensively discuss the potential of “organ-on-a-chip” devices and their applications in this field. Overall, this review sheds light on the most widely used models and methods, guiding researchers in the rational choice of strategies for studies of microbiota-gut-brain interactions.

## Introduction

Exploring the role of gut microbiota in human health and its alterations in disease development is a prominent field of research. This ecosystem is especially considered due to its involvement in gut-brain crosstalk following discoveries of association with various diseases. Studies of the microbiota-gut-brain axis have revealed associations between microbiota alterations and several human diseases, including immunological, neurodegenerative, and neuropsychiatric disorders.^1^ Gut-brain communication was initially thought to be limited to the effects of the nervous system on the pathophysiology of the gastrointestinal (GI) tract and vice versa. To date, it is known that the gut microbiome plays an essential role in neurodevelopment and communicates with the brain via the vagal nerves to relay the peripheral signals from the gut to the brain. The brain can also modulate gut physiology, taking the efferent route of the microbiota-gut-brain axis, like alteration of intestinal permeability by stress mediators.^2^ However, the microbiota-gut-brain axis is now considered a potential target for interventions in numerous neuropsychological diseases and GI inflammatory disorders, such as inflammatory bowel disease (IBD) and irritable bowel syndrome (IBS).^3^ The microbiota-gut-brain axis has been shown to play an essential role in the modulation of the brain and behavioral activities of the host from the intestinal microbiota.^4^ Indeed, within the gut mucosa, immune cells, microorganisms, and neurons interact to regulate workflow in the intestine and modify brain activities and behavior. It has been argued that the metabolites produced by intestinal microorganisms – involved as components of the mucosal immune system – and gut neurons are the major players in the interactions between the gut and brain. However, several key issues remain to be resolved in this field, including the choice of techniques for investigating how these molecular interactions occur and the axes that mediate them. Significant *in vitro* and *ex vivo* systems have also been developed to study the microbiota-gut-brain axis’s metabolomic and secretary aspects. Many of the diverse experimental designs created are based on the role of the intestinal microbiota and its influence in the modulation of GI pathophysiology and the brain’s interaction pathways^5^; beyond this, it appears the liver-brain axis and maybe other organs associated with the microbiota and nerves such as skin or lungs. The mucosal immune system plays a role in microbiota-gut-brain interactions, and many of the methodologies used are based on neuro-immune interactions. We systematically reviewed recent advances in techniques and models for studying microbiota-gut-brain axis interactions. First, we provide an overview of the available *in vivo* animal models and present *in vitro* and *ex vivo* techniques used on experimental platforms to study the microbiota-gut-brain axis. Finally, we propose ways to improve methodologies that provide us with a clearer perspective for future investigations.

## Materials and methods

**Figure 1.**
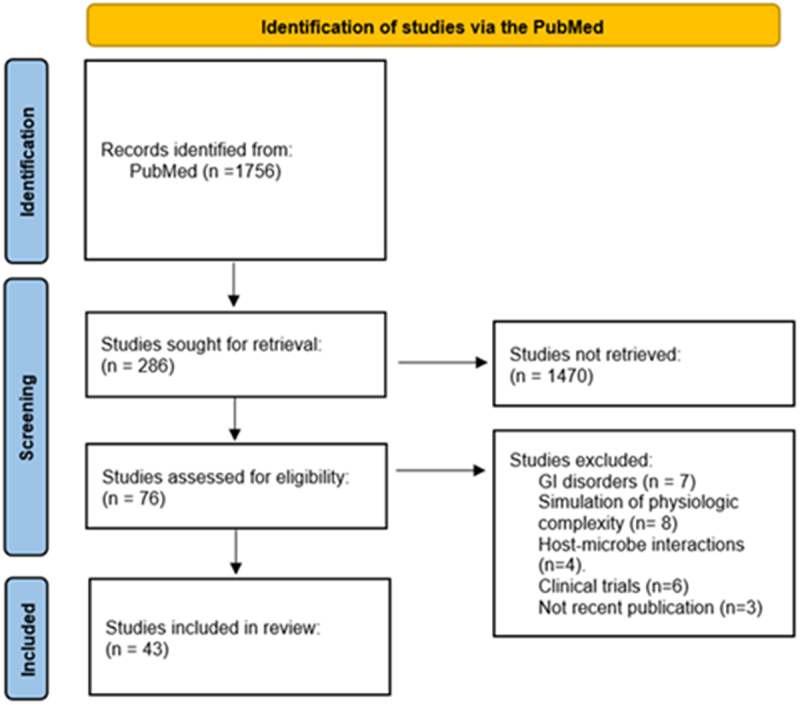
PRISMA flowchart, showing literature search.

Literature search strategies and selection criteria: We performed a systemic literature review on PubMed until September 2023 according to PRISMA guidelines. The terms used included “microbiota-gut-brain interactions”, “microbiota-gut-brain axis”, “animal models for studying gut-brain axis”, “gnotobiotic mouse gut-brain-axis models”, “gut-on-a-chip model”, “microbiota-gut-brain axis”, “organ-on-a-chip”, “3D culture systems”, “gut microbiome”, and “zebrafish”. The flow-chart diagram (Figure 1) includes publications based on title and abstract reading, techniques, and methods that have specifically improved or been proposed for studying microbiota-gut-brain axis interactions. Therefore, we excluded techniques that mimicked the intestinal environment and physiology for other purposes, as many publications deal with intestinal models that have been improved but have not yet been used or proposed for use in studies of microbiota-gut-brain interactions. Forty-three studies are included in this systematic review.

## Overview of animal models

**Figure 2.**
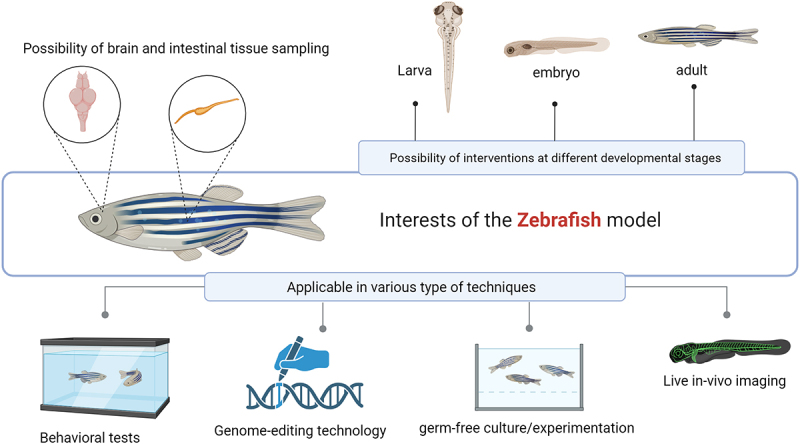
Figure 1: a schematic representation of in-vitro, ex-vivo, and in-vivo models in studying microbe-host interactions. (a) microbial culture approach for the analysis of microbe-derived metabolites. (d) advantages and characteristics of zebrafish as an animal model in microbe-host interaction studies of the gut-brain axis. Created with BioRender.com.

Various animal species have been used as models in studies of the microbiota-gut-brain axis. Figure 2 presents the multiple techniques used to study materials from animal models. In mammalians, rodents and, in particular, mice have been widely used as models to first study the role of the microbiota on physiology.^6^ However, several other types of animals, including Brandt’s voles,^7^ tree shrews,^8^ frogs,^9^ honeybees,^10^ wild house sparrows,^11^ laying hens,^12^ pigs,^13^ rhesus macaques,^14^ and Japanese quails,^15,16^ were also employed in the frameworks of microbiota-gut-brain axis and provided notable results for their specific characteristics of modeling human disease conditions. The nematode *Caenorhabditis elegans* emerge as a valuable model for dissecting the molecular basis of microbiota-gut-brain interactions.^17,18^ Studies of microbiota-gut-brain communications in animal models, particularly rodents, are based on the effect of the microbiota and the signals induced in either a simulated model of a particular disease or physiological behaviors of the animals concerned, such as emotional behavior, learning, and memory, responsiveness to stress, social behavior, and autism-like characteristics. Horvath et al. found that *Bacteroides ovatus* generates metabolites acetic acid, propionic acid isobaric, and isovaleric acid, which is associated, when inoculated to germ-free or gnotobiotic mice, with the production of GABA, detected in fecal samples. This GABA could then be associated with communication with the host and used by capture for the nervous system functioning.^19^ The gold-standard methodology to understand the microbiota of the brain is the usage of germ-free animals. Various species are grown in a sterile condition called axenic. These conditions guarantee that the inoculation of microbiota from the same or other species, in healthy or disease conditions, is associated with changes manifested by the model. Gnotobiotic models are animals with known and controlled microbiota, enabling the study of known bacteria strains. In other words, a germ-free model, inoculated with a known microbiota, is considered a gnotobiotic. Using a germ-free mouse model, Engevik et *al*. showed that *Bifidobacterium dentium* mono-associated colonization modified their behavior by modulating 5-hydroxytryptamine-5-HT-receptor expression in the gut and the brain. The authors found that *Bifidobacterium dentium* colonization partially restored 5-HT-dependent behavior, including abnormal anxiolytic changes observed in germ-free mice.^20^ Other researchers using germ-free mice demonstrated that gut microbiota colonization from different rodent species with distinct foraging strategies influenced the host diet selection behavior.^21^ Li *et al*. suggested that Rifaximin, a non-absorbable antibiotic, can ameliorate depressive-like behavior in rats by regulating the abundance of fecal microbial metabolites, such as SCFAs, and microbial functions by depleting some bacterial strains.^22^ Furthermore, using the gnotobiotic mice model, researchers showed that early colonization with complex microbiota was beneficially effective in rescuing behavioral abnormalities observed in germ-free mice.^23^ Germ-free mice that have received fecal microbiota transfer from a patient suffering from schizophrenia and intensive mental trouble present behaviors that could be apparated to human schizophrenia. This suggests that the microbiome could be relevant to the pathology of this disease.^24^ For straightforward reading, we choose to present in Table 1 the list of available animal models, their study designs, and the expected outcomes of the microbiota-gut-brain interaction pathways studied.

**Table 1.** Mammal models used in the study of microbiota-gut-brain interactions.

Animal model	Study design	Outcomes	Suggested microbiota-gut-brain interaction pathways	Ref.
Germ-free (GF) male C57BL/6 mice^25^ 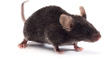	Fresh stool samples were collected from alcohol use disorder (AUD) patients and, using established protocols, transferred to colonize GF mice, followed by alcohol preference and drinking experiments and microbiota and SCFA analysis.	Their results showed that fecal transplantation from humans to GF mice reduced ethanol acceptance, intake, and preference, with lower murine alcohol intake and preference in post-transplant mice.	They suggested that these effects (reduction in alcohol craving and intake) are linked with multiple factors, including specific microbial genera, reflecting the importance of the microbiota-gut-brain axis.	25
Male ICR mice^26^ 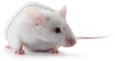	This model of neurodegenerative disease was established by treating mice with scopolamine. Passive avoidance tests were performed to observe and quantify cognitive impairment.	Mice subjected to pretreatment with neuromide had better cognitive function.	It was suggested that gut microbial metabolites (neuromide) might affect brain health via the endocannabinoid system.	10
Wild type (C57BL/6), transgenic (e.g. Fos^GFP^), and gnotobiotic (GF C57BL/6) mice^27^ 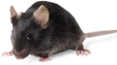	Mice were housed under a 12 h light-12 h dark photoperiod with free access to food and water. They were crossed into the animal facility to obtain various transgenic and gnotobiotic strains.	Gut microbiota influences on the enteric neurons were characterized and shown to modulate gut-extrinsic sympathetic neurons.	It was concluded from the results that the microbiota controls gut-extrinsic sympathetic activation through a microbiota-gut-brain circuit.	12
Male Wistar and Fischer rats and pigs^28^ 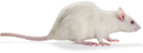 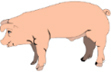	Rats received phenyl-γ-valerolactone, a critical microbial metabolite of phenolic flavon-3-ols (F3O). Also, rats received oral supplementation with lyophilized red grapes, and one group of pigs received a cocoa powder supplement to ensure F3O intake. After sacrificing, their brain tissues were collected and checked for F3O metabolites by UHPLC and MS/MS.	Colonic metabolites of F3O were detected in the brain tissues in both animal models after treatment with the metabolites mentioned above as a dietary supplement. These metabolites were, therefore, able to permeate into the brain.	Based on these results, it was argued that phenolic compound-rich foods may exert neuroprotective effects by influencing the gut microbiota acting via the microbiota-gut-brain axis.	13
Male Swiss CD-1 mice^29^ 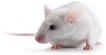	Cognitive impairment models were generated by amyloid β (Aβ) induction, and donepezil was used for memory recovery, tested with the Maris Water Maze (MWM) test. Changes in brain and gut metabolites and fecal microbiota were performed by Metataxonomic and metabolomics investigations.	For example, the relative abundance of Verrucomicrobia within the microbial community was higher in Aβ+donepezil-treated mice than with Aβ alone. Metabolic pathways of amino acid and sugar were affected by the Aβ and donepezil treatments in the brain and gut, respectively.	It was suggested that changes to the gut microbiota might influence the induction and attenuation of Aβ-induced cognitive dysfunction via the microbiota-gut-brain axis.	14

Researchers in the field of neurodegenerative diseases use the canine model. This is because it presents parallel features of brain aging. It represents a better translational model because of the environment, intestines, and habits closer to humans. Interestingly, in MGBA research, the canine diet is closer to the human diet than the mouse diet and is an adaptive genomic to starch-rich diet due to domestication. This is an important feature to consider when analyzing the effect of diet on the gut microbiome and its impact on the brain.^30^ A study linked intestinal bacteria from microbiota (fewer abundance of *Fusobacteria* and *Actinobacteria*) with memory faculties.^31^ Interestingly, studies have shown that aggressive behavior is associated with the bacterial genus, which is related to experienced anxiety. It was also shown, as in mice, that chronic stress induces anxiety related to specific microbiota bacterial composition.^32^ Probiotic therapeutic studies were conducted to address the veterinary treatment of aggressivity and anxiousness of dogs and showed promising beneficial effects.^33^ Metagenomic studies in dogs have shown a correlation between aggressive behavior and specific bacterial taxa of the intestinal microbiome and may be a predictive factor of evolution to aggressive behavior.^34^ These features rank dogs as models for MGBA studies, especially in studies concerning diet influence on behavior or neurodegenerative diseases.

The linkage between gut microbiota and behavior was tested in Rhesus Macaques (*Macaca Mulatta*). Founded relationships are consistent with those in humans. This opens a broad field of study to assess the neurobehavioral effects of methods to modulate the gut microbiome in complex behavioral features.^14^

The stress-induced model of piglets by maternal separation was used in a study investigating the effects of alkaline water on diarrhea induced by weaning stress. The hypothesis of an intestinal improvement through the hypothalamic-pituitary axis mediated by a modified bacteria ecosystem was tested. This study shows the reduction of cortisol and haptoglobin, according to induction of signal at the epithelial level that secondly modified the MGBA, conferring diarrhea resistance.^35^ These initiatives demonstrate a possible application of MGBA research beyond the widely used mice model.

Among non-mammalian models, zebrafish appear to be the widest-used model of microbiota-gut-brain interaction studies, and features are presented in Figure 2. The three main appearing benefits of zebrafish as a model are: (i) genome-editing in zebrafish is easy, allowing the performance of genetic manipulations; (ii) zebrafish can be used for live *in vivo* imaging of host-bacteria interactions to monitor the activities of immune-signaling components among other possibilities, and (iii) protocols for germ-free experiments are well-established for this species. Moreover, despite substantial differences in taxonomic composition, the microbiomes of zebrafish and humans have similar abundances of functional pathways.^36^ A Germ-free zebrafish model was studied using caffeine to trigger a neural hyperactivity model. The effect of melatonin as a probiotic agent was assayed on neurotransmitter production disorders compared to germ-free conditions. Disorders of brain neurotransmitter production (DA, γ-GABA, and 5-HT) caused by caffeine were improved by melatonin treatment, associated with the restoration of intestinal microbiota, compared to the maintenance of the axenic condition. This suggests that the healthy intestinal microbiota, modulated by melatonin, improves neurotransmitter secretion disorders.^35^ *Lee et al*. present zebrafish as an excellent animal model for microbiota-gut-brain axis studies of its small body, genomic/physiological similarities to humans, and its suitability for chemical screening *in vivo*. Zebrafish can mimic various human diseases: autism spectrum disorder (ASD) and Alzheimer’s disease (AD).^30^ Features of the GI are primarily conserved between mammals and zebrafish, except for acidic stomachs. The cell types in the zebrafish GI play a crucial role in sensing environmental stimulation and transmitting this information to other organs, including the brain. The zebrafish’s enteric nervous system (ENS) consists of enteric neurons, a submucosal/myenteric plexus, associated glia, and muscle layers, together with neurons capable of secreting neurotransmitters similar to those found in mammals. The zebrafish ENS has been shown to regulate intestinal motility and to mediate connections between the intestine and the central nervous system (CNS). The zebrafish immune system has also been shown to be highly similar to that of mammals, with most of the immune cells present in mammals, including macrophages, neutrophils, and B and T lymphocytes also identified in zebrafish. The CRISPR/Cas9 system has been validated to achieve gene knockouts effectively in zebrafish in vivo. Several transgenic zebrafish lines have been established for cell tracking in vivo to investigate communication between the gut microbiota and the brain. The host response to gut colonization by the microbiota has been reported to be similar in zebrafish and mammals, making it possible to transpose some zebrafish data to humans.^37^ Based on the microbiota-gut-brain axis, Chen et *al*. assessed the effects of isorhynchophylline (a traditional Chinese medicine used to treat addiction) on morphine dependence in a zebrafish model of morphine-induced addiction. The authors provided evidence that the impact of isorhynchophylline on morphine addiction is related to gut microbiota.^38^ Such studies could not only lead to diagnostic/therapeutic strategies in such addicted conditions but also support the use of the zebrafish model in studying the microbiota-gut-brain axis further.

Animal models still have known limitations.^30^ First, the animals used may have limited physiological relevance due to interspecies differences.^39^ Another major problem is the need for an accurate animal model capable of reproducing human enteric nervous system (ENS)-microbiome interactions faithfully.^30^ No specific animal model appears optimal for studies of microbiota-gut-brain interactions, particularly regarding the feasibility of transposing outcomes from these models to human pathophysiology. Finally, using animal models induces more and more ethical concerns that are stimulating researchers to turn to in vitro techniques that are more and more relevant in microbiota-gut-brain axis modeling.

### In vitro approaches

In vitro techniques use cells from immortalized lines or growth from explants. These devices enable a high precision of the biological and molecular mechanisms involved in the microbiota-gut-brain axis. We detail techniques from the simplest to the most complex developed.

#### 2D cellular studies

These cell-based study methods have several advantages, including cost-effectiveness, ease of handling, and robustness across different cell types. However, one major problem with 2D culture systems in microbiota-gut-brain studies is that these systems need more of the human body’s biological, mechanical, and topographical complexity.^40^ In particular, the neural network cannot be accurately represented in 2D culture systems. *Seo* et al. validate a 2D cellular model suggesting that gut microbes might interact with the brain through the endocannabinoid system, exerting a neuroprotective effect via the microbiota-gut-brain axis. They used a culture system in which PC-12 cells were grown in a 96-well plate. The cells were then treated with neuromide, a compound with a structure bioidentical to specific commensal bacterial metabolites, which functions as an agonist of the endocannabinoids CB1 and GPR119. Cell viability was tested after exposure to reactive oxygen species (ROS) and showed a significantly increased cell viability.^26^ On the other hand, *in-vitro* culture models of intestinal microbiota, such as the SHIME (Simulator of the Human Intestinal Microbial Ecosystem) model, have also shown the potential to be applied not only in the field of gut microbiome research but also in the study of microbe-host-interaction. This model mimics the entire GI tract and allows inoculation of the gut microbiome from different targets: diseased patients, healthy individuals, and animals (pig, dog).^41^

#### Organoid culture system

**Figure 3.**
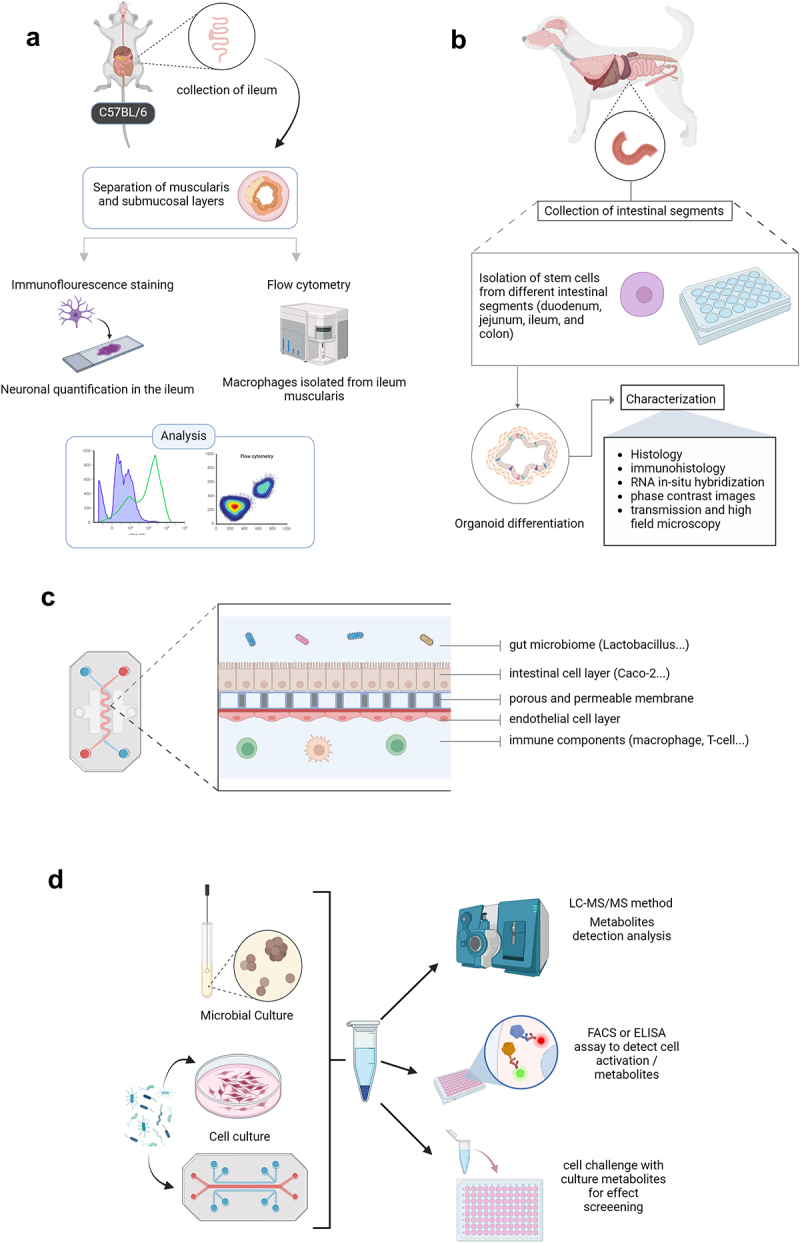
A schematic representation of an organ-on-chip model and the fundamental compartments of a gut-on-chip model. (a) ex-vivo intestinal isolation and technical applications to study cellular features of the intestinal tissue. (b) schematic presentation of 3D organoid culture system and technical potential application to understand microbiota-gut-brain interaction from dog intestine. (c) schematic representation of a gut-on-chip model that mimics intestinal microenvironment. (d) a technical approach is used to evaluate in vitro conditions and the effects of metabolites. Created with BioRender.com.

Substantial advances have been made in intestinal *ex vivo* culture by developing organoid culture systems. Yassachar *et al*. developed a microfabricated 3D organ culture system capable of preserving the average multicellular composition of the mouse intestine. Intact intestinal tissue from the mouse is connected to the input and output of the chamber, coupled with pumps to control the flow of the medium within the lumen and in the external medium chamber. This system allows us to model the interaction between intestinal cells, the immune system, microbes, and nutrients. They exposed this system to two different microbes and showed that it could reproduce the induction of RORg^+^ Treg cell populations and Th17 by *C. ramosum* and segmented filamentous bacteria (SFB), respectively. Indeed, they showed that the sensory neurons were activated by microbes associated with RORg^+^ Treg induction. They concluded that differential engagement of the enteric nervous system might be involved in pro- or anti-inflammatory responses to microbes.^42^ Trapecar et *al*. developed a mesofluidic culture system of gut-liver-brain interactions in the context of Parkinson’s disease (PD). Their platform is based on three microphysiological systems (MPSs) – gut/immune, liver/immune, and cerebral/immune systems – linked via the culture medium. They used HC176 colon organoids from non-diseased tissue biopsy specimens, and they seeded the system with human monocyte-derived dendritic cells and macrophages as innate immune system components of the gut. Liver MPSs were prepared from human primary hepatocytes from a single donor, and Kupffer cells were purchased. Coculturing neurons, astrocytes, and microglia in 24-well Transwell inserts established the brain MPSs. The circulatory system was irrigated with a serum-free culture medium supplemented with circulating CD4^+^ Treg and T_H_17 cells. Finally, purified short-chain fatty acids (SCFAs) were added as microbial metabolites. This system comprises pneumatic and mesofluidic plates separated by a polyurethane membrane to form a pumping manifold. The interactions between brain MPSs from healthy controls and MPSs of the gut-liver axis occurring in the presence of circulating Treg and T_H_17 cells had beneficial effects on the phenotype of the brain MPSs by increasing the expression of genes associated with the maturity of neurons, astrocytes, and microglia. They also observed that microbiome-associated SCFAs increased the expression of disease-associated pathways in PD. In isolation, samples from the cerebral MPSs were subject to metabolite extractions and analysis by reversed-phase ultra-performance liquid chromatography – tandem mass spectrometry (RP/UPLC-MS/MS).^43^ In an ex vivo culture system of duodenal samples from dogs with chronic enteropathies, Sauter et *al*. investigated the influence of probiotics on mRNA and protein expression levels of cytokines. Their results showed the beneficial effects of probiotics on cytokine expression and had an immune-modulating impact on intestinal inflammation by contributing to the reduction of inflammation.^44^ Ahrends et al. described how they isolated the myenteric and submucosal plexus intestinal layer from a mouse (C57BL/6) GI tract (Figure 3a). This layer contains an extensive network of enteric neurons, and thus, such methods could provide useful ex vivo experimentations of microbe-host interactions.^45^ Chandra et *al*. have developed a canine GI 3D organoid system model that is not only applicable to intestinal diseases in dogs and humans but will also help to investigate host-microbe interactions (Figure 3b).^46^ Brain organoid in vitro systems are also developed from human pluripotent stem cells, with several possible applications, including studies of neurological phenomena.^47^ However, the applications of brain organoids in the field of the microbiota-gut-brain axis require further investigation. Lack of critical components of in-vivo intestine like microflora, immune system, vascular and nervous systems is one of the main limitations of 3D organoid models, coupled with lack of a lumen, which results in the diffusion of intestinal secreted metabolites, like mucin, into the culture media. Another limitation of organoid systems growing in a 3D extracellular matrix is their variability in size, shape, morphology, and localization from one to another, making it difficult to achieve real-time monitoring.^48^

#### Organ-on-a-chip platforms

Microfluidic platforms combine lab-on-a-chip technology with 3D organotypic cultures to recreate the pathophysiological complexity of the microbiota-gut-brain axis, commonly called organ-on-a-chip (OoC) models. OoC models include different culture channel surfaces and fewer media requirements.^30^ They are designed to represent a single organ or as more complex multiorgan-on-a-chip platforms. They can provide a very thin culture chamber (millimetric dimensions), facilitating the continuous perfusion of the culture medium. They are accessible for imaging and quantitative assays, as sufficient cells can be harvested. OoC devices can also minimize functional aspects of pathophysiology in the tissues, making it possible to evaluate therapeutic agents and their effects on the tissue concerned. They can also lower the cost of research while increasing throughput over that achieved with animal models, thereby decreasing ethical concerns.^49^ These models can potentially model the microbiota-gut-brain microenvironment accurately, reproducing the physiological features observed *in vivo*.^30^ Platforms and devices of this type mimic *in vivo* organ physiology and *in vitro* function in a controlled environment.^39^ Figure 3 presents different schematic compartments of an organ-on-chip model for studying host-microbe interactions in vitro. OoC models are composed of multilayered and different compartments receiving cultures of endothelial cells, epithelial cells, macrophages, and dendritic cells.^50^ Most gut-on-chip models initially incorporate the intestinal microenvironment, including gut microbes, epithelial cells, and immune cells (Figure 3c). OoC is designed in various sizes and shapes. However, they all contain hollow channels lined by living cells cultured under fluidic flow, and OoC can be designed as a single and or multi-organ-on-chip. At the same time, it can also include multiple micro-physiological systems (MPSs), including the gut, liver, immune, and cerebral MPSs. Gut-on-chip models have different application areas, including their use in studying microbe-host interactions.^51^ Kim et *al*. described a biomimetic human-on-a-chip microdevice composed of two microfluidic channels separated by a porous membrane and coated with an extracellular matrix (ECM). The intestine structure is mimicked by human intestinal epithelial Caco-2 cells, where a fluidic flow recreates the gut microenvironment. Under those conditions, a columnar epithelium develops to recapitulate the intestinal villi. In addition, this model allowed the co-culture of *Lactobacillus rhamnosus* to be a normal intestinal microbe.^52^ Microbiota-gut-brain axis OoC requires microchannels (for perfusion and the establishment of biological gradients), microchambers (for the spatial separation of different cell types or tissue formations), extracellular matrix components (to ensure accurate representation in three dimensions), and electroactive compartments for stimulation and recordings.^36^ Thanks to microfluidic isolation, sampling the specific supernatant of cell culture is possible and enables targeted -omics analysis. Gabriel-Segard *et al*. designed a microfluidic device and used it to show that bacterial particles (Lipopolysaccharide) were able to generate electrophysiological activity in glutamatergic neurons mediated by immune cells (MoDC).^53^ This promising model could accelerate efforts to develop a gut-nerve-on-a-chip model for studying secretory aspects of the microbiota-gut-brain axis. The MINERVA project consists of a microbiota-gut-brain-engineered platform for evaluating the impact of intestinal microbiota on brain functionality using a multiorgan-on-chip. It consists of five organ-on-a-chip devices corresponding to the gut microbiota, gut epithelium, the immune system, the blood-brain barrier, and the brain. Each device is connected to the next via a microfluidic pipeline through which the culture medium can flow under positive pressure.^49^ Sampling and cell perfusion can be performed in each of the compartments. The first device mimics the gut mucus inoculated with gut microbiota, and the next is seeded with gut epithelial cells. The third device contains host macrophages and lymphocytes to represent the immune system, and the fourth consists of two mirror monolayers of endothelial cells and astrocytes representing the blood-brain barrier (BBB). Finally, the brain on-a-chip device consists of a 3D hydrogel matrix mimicking the brain extracellular matrix, into which neurons, microglia, and astrocytes are implanted. All the devices except those corresponding to the brain have microporous membranes to support cell adhesion and allow the secretome to pass into the lower part of the culture chamber without mixing the different media. One perspective for improving OoC models is the generation of so-called “body-on-chip” platforms integrating several OoC devices. Fusco *et al*. proposed the development of a multiorgan-on-a-chip platform for investigating the role of the microbiota-gut-brain axis in the context of epilepsy.^36^ Last but not least, we shall highlight here the challenges and limitations of OoC models that could be useful for the future improvement of such models. One main challenge is to move to the next level of OoC models to demonstrate the equivalence and/or superiority of these techniques to animal models.

#### Analytical platforms for metabolomic studies of the microbiota-gut-brain axis

Microbial metabolites have several different roles in microbiota-gut-brain crosstalk by triggering immune system activation or influencing the development of neurodegenerative disorders. Therefore, well-balanced metabolite production by the microbiota is essential for host health, and any changes to microbial metabolism may influence microbiota-gut-brain interactions. For this reason, metabolic analyses of microbiota are a valuable tool for studies of the microbiota-gut-brain axis (Figure 3d). The most widely used analytical platforms for metabolomic analyses of the microbiota-gut-brain axis include liquid chromatography coupled with mass spectrometry (LC-MS), which can determine the levels of many organic acids, such as SCFAs, bile acids, and their derivatives. Gas chromatography coupled with mass spectrometry is also a versatile technique for metabolite determinations. The samples and matrices used for metabolomic analysis include stools, urine, plasma, serum, cerebrospinal fluid, intestinal biopsy specimens, and brain tissue.^1^ As microbiota-gut-brain axis studies involve multiple soluble molecules, highly varied omic methods are required to identify biological pathways. In an analysis based on a gnotobiotic animal model, intestinal organoids, bacterial cultures, and stool sample, Horvath et *al*. performed a targeted liquid chromatography-tandem mass spectrometry (LC-MS/MS)-based metabolomic analysis to investigate SCFAs, intestinal and brain neurotransmitters. They showed that *Bifidobacterium dentium*, a commensal bacterium present in the gut, produces GABA (gamma-aminobutyric-acid) and that colonization of the intestine by this bacterium is associated with higher gut serotonin levels. They also showed that *Bifidobacterium ovatus* secretes indole-3-acetic acid *in vitro*, thereby increasing the levels of this molecule in the cecum and feces in an animal model. They demonstrated that this bacterium (*B. ovatus*) affects CNS gene expression, microglial maturation, and mouse behavior and can modulate immune cells, conferring protection in an animal model of colitis.^54^

#### Mechanosensory probe, electrophysiological recordings, and neuroimaging

Mayeli et *al*. developed a minimally invasive mechanosensory probe to target the perceptions of the gastrointestinal system and neural responses to gut sensations via the ingestion of a vibrating capsule. The design of their study was inspired by signal theory, and it combined the mechanosensory stimulation of gut signals with measurements of gut sensations, electroencephalogram (EEG) and electrogastrogram (EGG) recordings, and the recording of other peripheral physiological signals. Gastrointestinal stimulation was achieved in healthy individuals by ingesting an orally non-biodegradable vibrating capsule. The capsule’s vibration was detected with a stethoscope, and electrophysiological recordings were performed with an EEG system. The pre/post-processing data were then analyzed with Brain Vision Analyzer-2 software. This approach made it possible to identify signatures of gastrointestinal perception and differential effects in the brain according to the strength of stimulation. The authors suggested that this approach would be helpful for investigations of microbiota-gut-brain interactions in individual humans.^55^ Overall, according to many studies, combining the data from the gut (microbiota) with the methodologies like EGG/EEG,^56^ noninvasive electric recording system,^57^ and neuroimaging modalities such as MRI (Magnetic Resonance Imaging) have provided insights into the understanding of microbiota-gut-brain interactions.^58–61^

## Discussion

Various techniques and models have been used to study the microbiota-gut-brain axis by investigating the components of this crosstalk. Animal models have been widely used to determine the effects of the microbiota on the microbiota-gut-brain axis, whereas diverse *in vitro* models have been used to reproduce various aspects of the pathophysiological microbiota-gut-brain microenvironment. Mice are the most widely used animal model in this context. Still, other animal models are emerging, including the zebrafish, which is considered particularly promising for use in studies of the microbiota-gut-brain axis – germ-free animal experimentations are still the gold standard for studying microbiota-gut-brain interactions.^62,63^ Researchers have suggested the employment of multi-animal species in the future use of animal models in the field,^64^ as microbiota-gut-brain social behavioral features are not realizable through *in vitro* models. However, it is too early to conclude that the modeling strategies used in this field can address all the questions raised concerning microbiota-gut-brain interactions, and we may still have some way to go before a more relevant modeling strategy is found. Some of the systems developed to date require manufacture in a complicated multi-step process that is time-consuming and labor-intensive. It also remains difficult to determine the optimal conditions for the coculture of the elements of the microbiota-gut-brain axis, particularly in 2D and 3D models, in which various components are required. There is also yet to be a definitive method for tracing the dynamics of *in vitro* microbiota-gut-brain models.^39^ Despite the challenges outlined above, *in vitro* models are considered promising based on the evaluations of several types of platforms. OoC devices are considered promising tools with many advantages for studies of the microbiota-gut-brain axis. Firstly, OoC platforms allow cell culture in single chambers connected by chip-based microfluidic channels to promote communication. Secondly, it is possible to control the microenvironment’s spatial and temporal features and create more physiologically relevant complex systems. It also allows setting medium flow rates (exchange of nutrients and metabolites), simulating cell growth, proliferation, and differentiation, applying mechanical forces (to mimic the physical microenvironment), and monitoring the operating parameters (oxygen, glucose concentration, pH). The miniaturization of these devices also makes it possible to decrease reagent volumes. Moreover, electric sensors can be integrated into these devices, making it possible to evaluate biological and biophysical parameters, such as transepithelial electrical resistance (TEER), and through micro-electrodes arrays (MEA), the electric activity within a neuron culture. Human brain imaging techniques have been used to explore possible interactions between the functions of the gut and those of the brain in certain neuropsychiatric disorders and to evaluate brain activities following the administration of commensal-fermented milk through cognitive function tests.^4^

Information and data from human studies on microbiota-gut-brain interactions still need to be made available, highlighting the need for approaches based, to a more significant extent, on pathophysiology and clinical data.^5^ Therefore, focusing on human diseases and neurological disorders as model candidates for studying microbiota-gut-brain interactions would be interesting. IBD patients have been widely investigated in studies of the microbiota-gut-brain axis, leading to several significant achievements in regulating inflammation by the vagus nerve or transmitting stress by the vagus nerve, intensifying by triggering inflammatory relapses of the disease. Liu et *al*. investigated correlations between IBD and changes in cerebral cortical structures, which can imply the existence of microbiota-gut-brain involvement at the organismal level. The authors suggested that magnetic resonance imaging (MRI) might be considered an additional screening option for IBD patients and that clinical patients with IBD prioritize long-term inflammation management, as changes at the organismal level can lead to functional pathologies.^65^ Drug addiction is a significant public health issue in many countries and drug-dependent individuals may serve as relevant candidates for the study of microbiota-gut-brain interactions in this context. The pathways and systems involved in the pathophysiology of such disorders include nutrient intake, mental health, and the immune system, all essential factors and elements of the microbiota-gut-brain axis. By contrast, fewer studies have been performed, and less extensive data are available concerning the electrochemical properties of the microbiota-gut-brain axis. So, it would be helpful to develop technical strategies based on the electrochemical properties of microbiota-gut-brain interactions. Focusing on specific topics or general analysis of techniques and methodologies in this field should strengthen research to improve our knowledge about the mechanisms underlying microbiota-gut-brain interactions.

Strategically, it is sensible to focus efforts on integrating cellular elements of human origin into the design of *in vitro* platforms whenever possible. Most of the *in vitro* systems in current use incorporate the coculture of cells, at least some of which originate from animals. Therefore, the results generated by such models may be similar to those expected from animal models, and their interpretation concerning humans would need to be revised. Finally, it would be helpful to compare a wide range of studies in different animal models and their outcomes to assess the similarities of results obtained from other animals regarding the molecular pathways of microbiota-gut-brain interaction identified and to determine the variability of these results.

## Conclusions

We provide information about some of the recently developed methods and models for microbiota-gut-brain studies, highlighting, in particular, the importance of zebrafish as an *in vivo* animal model and organ-on-a-chip systems as *in vitro* models. Our synthesis highlights the usefulness of both strategies in the field, as animal models cannot yet be entirely replaced, and *in vitro* models alone have limitations and cannot perfectly reproduce *in vivo* conditions. In the future, it might be helpful to perform a comparative review on *in vitro* models and strategies in which efforts have been made to simplify and isolate the elements of the microbiota, gut, or brain, comparing these systems with efforts to create much more complicated systems. This, in turn, might guide decision-making by researchers interested in designing models to study the microbiota-gut-brain axis.
